# Aerobic training improves blood-brain barrier and neuronal apoptosis in experimental autoimmune encephalomyelitis

**DOI:** 10.22038/IJBMS.2022.61671.13645

**Published:** 2022-02

**Authors:** Omid Razi, Abdolhossein Parnow, Iraj Rashidi, Nafiseh Pakravan, Seyed Ershad Nedaei, Robert W Motl

**Affiliations:** 1 Department of Exercise Physiology, Faculty of Physical Education and Sports Science, Razi University, Kermanshah, Iran; 2 Department of Anatomical Sciences, School of Medicine, Kermanshah University of Medical Sciences, Kermanshah, Iran; 3 Department of Immunology, School of Medicine, Alborz University of Medical Science, Karaj, Iran; 4 Department of Physiology, School of Medicine, Kermanshah University of Medical Sciences, Kermanshah, Iran; 5 Department of Kinesiology and Nutrition, College of Applied Health Sciences, University of Illinois Chicago, United States of America

**Keywords:** Aerobic training, Astrogliosis, Brain barrier dysfunction, Claudin-5, EAE model, Occludin

## Abstract

**Objective(s):**

Blood-brain barrier (BBB) permeability is central in multiple sclerosis (MS) pathophysiology, and exercise may improve BBB integrity. The current study investigated the prophylactic and/ or therapeutic role of aerobic exercise (EX) training on BBB integrity in experimental autoimmune encephalomyelitis (EAE).

**Materials and Methods:**

Forty female Lewis rats were randomly divided into four groups. The experimental groups included: no-EAE induction+ no-exercise (no-EAE+ no-EX), no-EAE induction+ exercise (no-EAE+EX), EAE induction+ no-exercise (EAE+ no-EX), and EAE induction+ exercise (EAE+EX). The no-EAE+EX and EAE+EX groups performed six weeks of progressive aerobic exercise training. GFAP, angiopoietin 1 (Ang-1) expression, tight-junction (TJ) proteins of claudin-5 and occludin were measured as components of BBB integrity and the rate of neuronal apoptosis was evaluated in hippocampi.

**Results:**

A significant increase in GFAP and Ang-1 expression (*P*<0.001) and conversely a down-regulation in TJ proteins (*P*<0.05) was found in the brains of the no-EAE+EX group compared with the no-EAE+ no-EX group. The expression of GFAP and Ang-1 proteins significantly increased in the hippocampi of the EAE+ no-EX group (*P*<0.001), whereas aerobic training (in the EAE+EX group) meaningfully reversed such increases (*P*<0.001). Besides, down-regulated TJ proteins and increased neuronal apoptosis induced by EAE induction (EAE+ no-EX group) were restored and reduced, respectively, by aerobic training in the CNS of the EAE+EX group (*P*<0.001).

**Conclusion:**

The provision of a six-week treadmill aerobic training buffered the detrimental effects of EAE on BBB integrity and consequently neuronal apoptosis.

## Introduction

Multiple sclerosis (MS) is a complex disease of the central nervous system (CNS), characterized by neuroinflammation, demyelination, astrogliosis, axonal loss, and neuronal death (1). The disease seemingly involves migration of activated peripheral T-lymphocytes against myelin-forming cells (oligodendrocytes) across the blood-brain barrier (BBB) (2). There is accumulating evidence that BBB disruption is an initial step of relapsing disease in both humans and animal models of MS, namely experimental autoimmune encephalomyelitis (EAE) (3, 4). 

The structure of BBB is normally maintained by multi-protein complexes, namely tight junction (TJ) proteins including claudin-5, occludin, and zonula occludens (ZO) (5). Apart from TJs, CNS resident cells, especially astrocytes, are involved in the maintenance of BBB integrity (5). These glial cells have extensive communication with adjacent cells including endothelial cells through end-feet (6, 7). With both MS and EAE, astrocytes convert to reactive astrogliosis which is highlighted by changes in morphology and function as well as expression of some genes such as glial fibrillary acidic protein (GFAP) (6, 8, 9). Astrogliosis is associated with increased BBB permeability through loss of physical connection between astroglial end-feet and endothelial cells (10).

Astrocytes are further involved in the maintenance of BBB through secretion of soluble factors such as angiopoietin-1 (Ang-1) (11, 12). In healthy conditions, released Ang-1 supplies anti-inflammatory and vascular repair effects, while in inflammatory conditions it has a pro-inflammatory role (13). Researchers have recently reported that astroglial responses are concurrently associated with initial inflammation and axonal damage as well as neuronal death (14). 

Exercise training may influence neuronal adaptation and morphological astroglia changes in size, complexity, and processes (15, 16). There is limited and inconclusive literature about reactive astrogliosis, as a critical factor in exacerbating BBB integrity, following exercise training (17, 18). Nevertheless, exercise may improve BBB integrity via preserving and expressing two tight-junction (TJ) proteins of occludin and claudin-4 in both the CNS and spinal cord of EAE (19). On the contrary, other documents reported that exercise as external stress may increase BBB permeability through reducing TJ proteins and increased level of GFAP as a marker of astrogliosis (20-22). 

There is an abundance of evidence on improving cognition function through exercise training-mediated alterations in hippocampal cell proliferation and apoptosis in both animal and human studies (15, 23, 24). Consistently, the results even in human subjects with MS indicated that aerobic training for 12 weeks surprisingly promoted parahippocampal gyrus volume and also reduced inflammation and as a consequence mitigated neurodegeneration (25). Accordingly, the hippocampus is a brain area of high pliability with exercise training and direct relevance for studying BBB permeability. 

The current research investigated the effect of aerobic exercise training on hippocampal components of BBB integrity and neuronal death in the EAE animal model of MS. We hypothesized that aerobic exercise training would result in decreased expression of GFAP on astrocytes and Ang-1 as well as apoptotic cells induced by EAE and increased levels of TJ proteins of BBB. 

## Materials and Methods

***Animals and ethical approval***

Forty female Lewis rats weighing 120–140 g and 6–8 weeks old were purchased from Daroupakhsh, (Pharmaceutical Mfg Co, Tehran, Iran). The animals were housed in standard and cleaned cages with controlled environmental conditions (21-22 °C, relative humidity 45–50%, 12 hr light/dark cycle). The food and weight of all animals were daily monitored. All procedures were performed in accordance with the ethical guidelines for the care and use of laboratory animals (principles of Helsinki) and approved by the animal care committee of Kermanshah University of Medical Sciences, Kermanshah, Iran (approval number: IR.KUMS.REC.1398.1130). 

***Experimental groups***

The first week was spent to accustom the animals into the new environment, and then they were randomly assigned into four groups: no-EAE+ no-EX (n=10) [no-EAE induction+ no-exercise], no-EAE+EX (n=10) [no-EAE + with exercise], EAE+ no-EX (n=10) [EAE induction + no-exercise], and EAE+EX (n=10) [EAE induction + exercise] at the end of the week. The second week was devoted to familiarization of main exercise training protocol. 

***Experimental training protocol***

**Figure 1:**
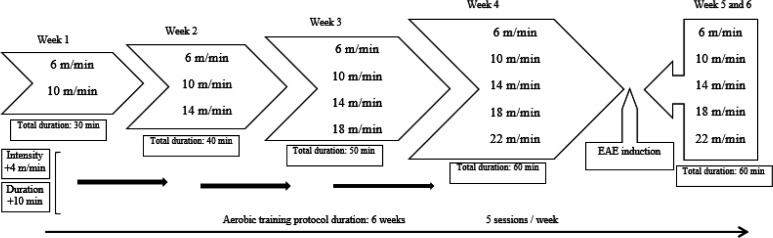
Schematic diagram of six-week aerobic exercise training. Each week consisted of some intensities. Week 1 started with 6 m/min for 5 min and terminated in 10 m/min for 25 min. Intensity +4 m/min means 4 m/min is increased to the next week and the same goes for other weeks as indicated by arrows. The time duration for week 1 is 30 min. Duration +10 min means the time added to the total time duration of the next week, as depicted by rectangles and black arrows on the left side

The familiarization period occurred over 4 consecutive days, such that the duration and intensity of days 1 to 4 were precisely similar to weeks 1 to 4 of the main training protocol (for more details refer to Figure 1). Otherwise, the electrical shock implemented to make animals run on the treadmill was 0.4 mv. The electric shock occurred with frequency of 3 to 4 times which usually lasted <1 sec during the session of the main training protocol. The main training protocol generally consisted of a six-week, five sessions/week progressive aerobic training on the treadmill (BX-TR5, BIOSEB In Vivo Research Instruments, France). Each session had a fixed phase of warming-up for 5 min and the final intensity of previous weeks was added to the following weeks which was implemented after the warming-up phase as an added 5 min phase. The training protocol included: week 1: 30 min (total time)= 5 min warm-up (6 m/min, ~46% VO_2_max) + 25 min (10 m/min, ~49% VO_2_max) (26), week 2: 40 min (total time)= 2×5 min + 30 min (14 m/min, ~53% VO_2_max), week 3: 50 min (total time)= 3×5 min + 35 min (18 m/min, ~55.2% VO_2_max), week 4: 60 min (total time)= 4×5 min + 40 min (22 m/min, ~59% VO_2_max). The 5^th^ and 6^th^ weeks were akin to week 4 in overall duration, intensities, and phases (Figure 1) (27). All training intensities were delivered on a motorized treadmill prior to initiation of the training program. EAE induction was induced after the ending of week 4 of the main exercise training protocol. Moreover, to simulate the stressful condition of exercise training on the treadmill, the rats of the non-EX group were placed on the treadmill lanes for the same duration as EX groups but did not receive the training stimulus. 

***EAE induction protocol***

The homogenate for EAE induction was prepared according to a previous study (2). Two hundred μl homogenate, which was prepared equally from 50% suspension of Guinea pig spinal cord (Department of Laboratory Animal Science, Tehran, Iran) and Incomplete Freund’s Adjuvant (IFA) (263910) (Difco, Germany) (1:1 v/v) containing 4 mg/ml *Mycobacterium tuberculosis* H3 RA (231141) (Difco Labs, Detroit, MI, USA) was injected to both sides of the tail base (100 μl per each side) in 20 out of 40 rats (EAE+ no-EX and EAE+EX groups). Following injection, the rats were transferred to the animal house to place them at the convenient circumstance and temperature to return consciousness. 

***Clinical signs scoring***

We measured clinical signs and weighed them daily during the 14 days post-induction (p.i) in a blinded manner. Clinical signs were scored according to a previous study (2), as follow: (1) complete tail paralysis; (2) mild paresis of hind limbs; (3) complete paralysis of one hind limb; (4) bilateral hind limb paralysis; (5) complete paralysis (tetraplegia) or death. The rats with clinical signs between two scores were given a half score.

***Tissue preparation***

Forty-eight hours following the completion of the training protocol on the 14^th^ day of p.i, the rats were anesthetized using ketamine and xylazine (3:1). Thereafter, the animals’ brains in each group were dissected by two different procedures in order to assess the markers of interest by western blot, immunohistochemistry (IHC), and terminal deoxynucleotidyl transferase (TdT) dUTP Nick**‐**End Labeling (TUNEL) techniques. Five animals in each group were sacrificed by cardiac perfusion of saline, followed by 100 ml of 4% paraformaldehyde in 0.1 M phosphate buffer saline (PBS), pH 7.4. The hippocampi were extracted and post-fixed in the fixative solution for 24 hr. Fixative hemispheres thence embedded to paraffin and coronal sections of 5-μm (3 sections per rat) were cut for assaying the interest markers by IHC and TUNEL staining. The sections were affixed on the slides and dehydrated overnight at room temperature. The remaining 5 rats from each group were anesthetized and then their brains were harvested on an ice-pack for evaluating by western blot technique. 

***TUNEL staining***

Neuronal apoptosis was detected by using TUNEL assay (TUNEL+ cells) (15). This evaluation was conducted utilizing an In Situ Cell Death Detection Kit (Roche, Pod, Germany) according to the manufacturer’s protocol. After deparaffinization, the sections (3 sections per rat) were placed in xylene (Sigma) for 10 min and then rehydrated in 90%, 80%, and 70% alcohols and washed in PBS solution. To make the rehydrated sections permeable, they were treated with 1 μg/ml proteinase K in PBS for 20 min at room temperature and then washed in PBS. Thereafter, the tissue sections were rinsed and incubated with 3% H_2_O_2_ in methanol for 10 min to block the action of endogenous peroxidase. In the next step, a TUNEL reaction mixture (50 μl) was added to the tissue sections and incubated for one hour at 37 °C in a humidified atmosphere. After being washed in PBS, the sections were evaluated by fluorescent microscope (Labomedtcs 400, United States). 

***Immunohistochemistry (IHC)***

IHC was conducted for Ang-1 and GFAP^+^ cells in the hippocampus by the method described before (15). Concisely, the coronal sections (5 μm, three sections per rat) were washed in PBS three times and then incubated in citrate buffer, pH 9.1 for 20 min at 70 °C. Antigens were retrieved by treating with 2 normal hydrochloric acids for 30 min. The sections were washed in PBS. 0.3% Triton was used to make the cells’ membrane permeable for 30 min and then the sections were washed in PBS. After being washed, 10% goat serum was added for 30 min to block the reaction of the secondary antibody as an additional background stain. Sections, then, were incubated with the primary antibodies (mouse monoclonal anti-GFAP antibody, Santa Cruz Biotechnology, Dallas, USA; polyclonal anti-Angiopoietin 1 antibody, Bioss, USA) diluted with PBS (1:100) overnight at 4 °C by maintaining the humidified chamber to prevent drying of tissue. After being washed in PBS 4 ×5 min, the tissue sections were incubated with diluted secondary antibodies (goat anti-mouse, Abcam, USA) (1:150) for one hour and a half in a dark room at 37 °C. The tissue sections were transferred from incubator to the darkroom, and after washing (4 times), DAPI was added to them as counterstaining for fluorescent detection. The sections were immediately washed in PBS. Finally, the sections were assessed using a fluorescent microscope (Labomedtcs 400, USA). 

***Western blot***

The expressions of claudin-5 and occludin were evaluated according to a previous study (28). Briefly, hippocampi were homogenized in radioimmunoprecipitation assay (RIPA) lysis buffer with cocktail protease inhibitors, and supernatants were skimmed. Protein concentration was identified by the Bradford procedure. The protein samples were separated using 10% sodium dodecyl sulfate-polyacrylamide gel electrophoresis (SDS-PAGE). The protein bands thence were transferred to the PVDF membrane (Bio-Rad, USA). After blocking with 5% skimmed milk powder solution at room temperature for 60 min, the membranes were incubated at 4 °C overnight with primary antibodies occludin (Monoclonal Antibody (OC-3F10), HRP, Invitrogen, USA), claudin-5 (rabbit-antibody unconjugated polyclonal, Biorbyt, UK), and recombinant glyceraldehyde-3-phosphate dehydrogenase (GAPDH, Abcam, USA), as per manufacturers’ instructions. After being washed 3 times with Tris-buffered saline and Tween 20 (TBST), the membranes were incubated with horseradish peroxidase (HRP)-conjugated secondary antibodies (Goat Anti-Rabbit, Abcam, USA) at room temperature for 60 min. After removing the secondary antibodies, the analyses were performed by assessing the intensity of protein bands which was quantified via Lab^TM^ Touch Software Version 1.2 (Bio-Rad, USA). 

***Statistical analyses***

The data were expressed as mean ± SD. The normality and homogeneity of data were examined with Shapiro-Wilk and Leven tests, respectively. Mann-Whitney U test was used to compare clinical scores between EAE and EAE+EX during 14 days p.i. Moreover, Friedman and Wilcoxon’s tests were performed to identify the significant differences within each group during repeated days. Mixed-factor repeated measure test was used to assay the effects of within (time) and between groups in weight status during 14 days p.i. The Dependent t-test was used for paired comparison of weight changes over time series, and multi-analyses of one-way ANOVA were conducted to precisely find significant differences between groups. Two-way ANOVA was used to assay the interaction between groups [EX × EAE] in each marker (astrogliosis, Ang-1, TJ proteins, and neuronal apoptosis). GraphPad Prism (GraphPad Software, San Diego, CA, USA) and SPSS software (IBM SPSS Statistics 21) packages were used to perform statistical operations. *P*<0.05 was considered a significant level. 

## Results

The obtained data were normal and homogenous, with exceptions for clinical scores (*P<*0.05), according to both Shapiro-Wilk and Leven tests (*P>*0.05), respectively. 

***Aerobic training delays clinical scores, and EAE induction causes weight loss***

**Figure 2:**
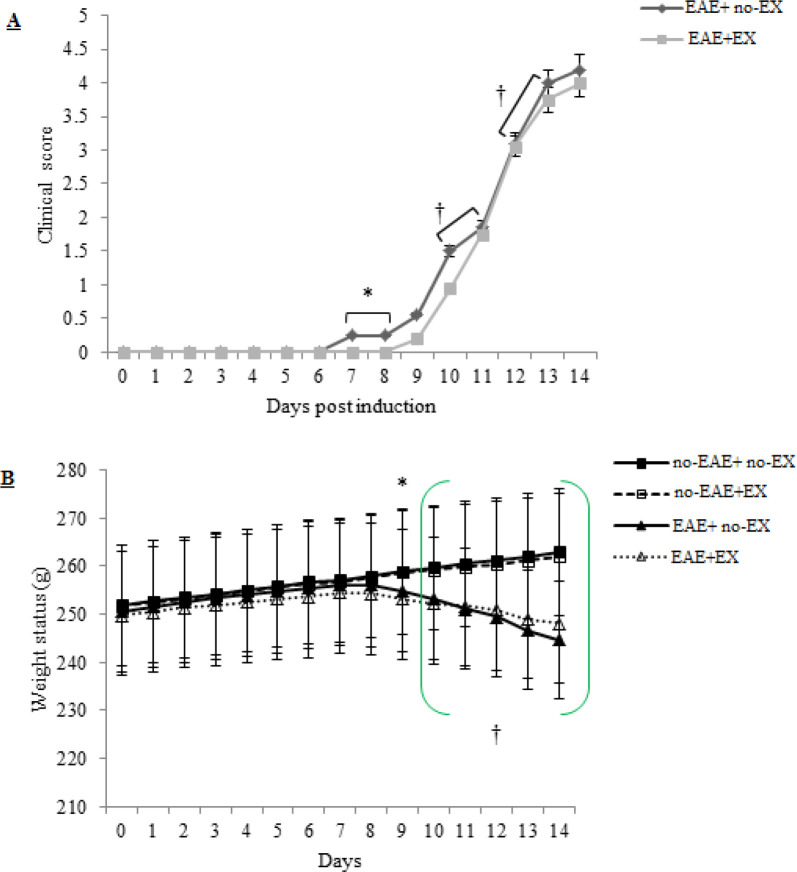
Clinical scores (A) and weight status (B) of Lewis rats implemented in both EAE-affected groups (EAE+ no-EX, EAE+EX) that are daily controlled during 14 days after immunization. (A) Aerobic training delayed clinical signs induced by EAE induction on days 7 and 8 and also attenuated the severity of clinical signs on days 11-12. (B) represents a significant weight reduction in EAE+EX on day 9 and weight loss among groups over days 10 to 14. (A) * Significant difference at *P*<0.05. (B) * Significant difference between EAE+EX and no-EAE+ no-EX groups on day 9. (†) Significant weight differences among no-EAE+ no-EX and EAE induction groups (EAE+ no-EX, EAE+EX) as well as among no-EAE+EX and EAE groups (EAE+ no-EX, EAE+EX) over days 10 to 14

EAE: experimental autoimmune encephalomyelitis; EX: exercise

As for the clinical scores, the obtained findings from the Mann-Whitney U test signified non-significant differences between EAE+ no-EX and EAE+EX groups in attenuating the severity of overall clinical scores (*P>0.05*), while the initiation of clinical scores was significantly delayed on days 7 and 8 in the EAE+EX group (r=0.48, large effect size) (*P<*0.05, Figure 2A). With respect to Friedman results, significant differences were observed in 14-day clinical scores in turn for EAE+ no-EX [X^2^(14, n=10) =129.90] and EAE+EX groups [X^2^(14, n=10) =131.90] which implies that the clinical scores are increased continuously during 14 days p.i for both groups (*P<*0.001). Furthermore, the findings of repeated measured Wilcoxon test illustrated that there were meaningful differences between days 10-11 and 12-13 p.i. in both EAE+ no-EX (r= 0.1, small effect size) and EAE+EX groups (r= 0.1, small effect size) (*P<*0.05); hence, the clinical scores in both groups on days 10 to 13 are markedly increased with a steeper slope. 

Regarding the resultant findings from the Mix-factor repeated measure test for weight loss (Figure 2B), there was a significant interaction [effect size= 0.79, large]. These results suggest that the changes in weight were significantly different between exercise training (no-EAE+EX) and EAE+ no-EX groups. The multiple analyses to find the differences between groups for every single day indicated that there was a significant difference between no-EAE+ no-EX and EAE+EX groups (*P<*0.003) on day 9 p.i. (*P<*0.001). Besides, more extensive differences were obtained in the no-EAE+ no-EX group compared with EAE+ no-EX and EAE+EX groups and also in no-EAE+EX compared with EAE+ no-EX and EAE+EX groups on days 10 to 14 (*P<*0.001). Thus, these findings reflect that the weight status procedures are different between exercise training groups (no-EAE+ no-EX and no-EAE+EX) and EAE induction groups (EAE+ no-EX and EAE+EX); the pattern of weight changes in both EAE groups (EAE+ no-EX and EAE+EX) and no-EAE groups (no-EAE + no-EX and no-EAE+EX) were downward and upward, respectively, during 14 days. The findings related to weight loss indicated that there was a main effect for time [effect size= 0.39, large] (*P<*0.001). In this interim, dependent t-tests were performed separately for every single group for 14 days. The multi-paired comparisons were statistically significant in no-EAE+ no-EX and no-EAE+EX groups during 14 days (*P<*0.05). Based on Figure 2B, such significant statistics within exercise training groups may disclose that animals’ weight tended to increase. The animals in EAE groups illustrated a significant weight loss during 14 days (*P<*0.05), with the exception of days 7–8 (*P>*0.05). In this context, it can be concluded that weight loss intensified when the clinical scores were going to be exacerbating. 

***Aerobic training increases GFAP expression but reverses the effects of EAE induction on GFAP***

**Figure 3:**
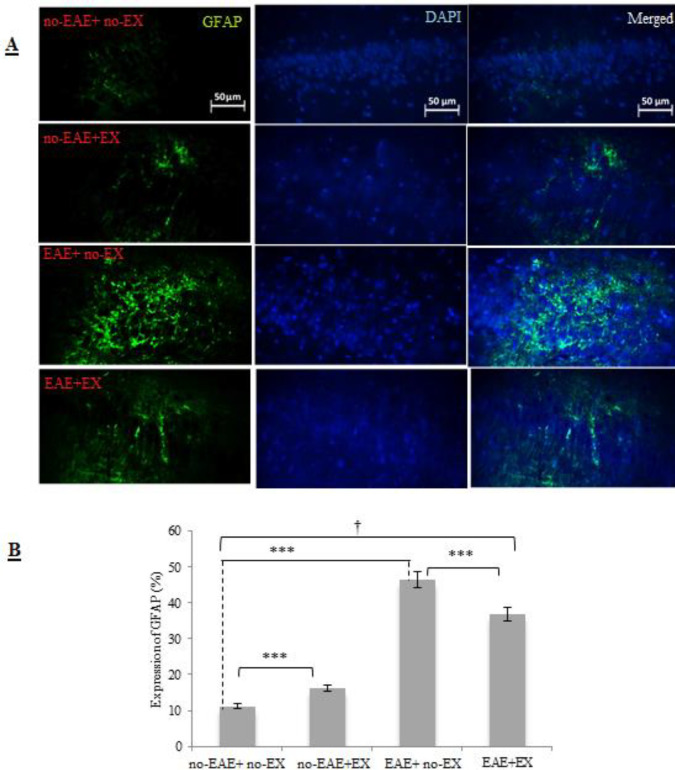
Level of GFAP expression on astrocytes (GFAP+ astrocytes) in four groups following a six-week treadmill aerobic training. A) Microscopic staining of GFAP+ astrocytes and B) Graph of % expression of GFAP protein based on statistical analysis of microscopic images by using IMAGE J software. Section (B) also shows aerobic training significantly promoted astrocytosis and also reduced astrogliosis induced by EAE induction. no-EAE+ no-EX, the group without exercise training and without EAE induction; no-EAE+EX, exercise training group; EAE+ no-EX, experimental autoimmune encephalomyelitis group without exercise, EAE+EX, the combined experimental autoimmune encephalomyelitis + exercise group. The data are expressed as percentages. *** Indicates statistical significance at *P*<0.001; † Indicates significant difference among groups at *P*<0.001

EAE: experimental autoimmune encephalomyelitis; EX: exercise; GFAP: glial fibrillary acidic protein

Two-way, 2 × 2 ANOVA indicated a significant interaction on GFAP expression on astrocytes as an indirect factor involved in BBB permeability (*P<*0.001, effect size = 0.92, large). Namely, different effects of EAE induction (EAE+ no-EX vs EAE+EX) with exercise training treatment (no-EAE+EX vs no-EAE+ no-EX) were observed on GFAP expression. As illustrated in Figure 3, meaningfully high GFAP expression on astrocytes was obtained in the no-EAE+EX group compared with no-EAE+ no-EX (*P<*0.001). There was a significant change in GFAP expression in EAE+ no-EX animals compared with the no-EAE+ no-EX animals (*P<*0.001). Additionally, GFAP expression was attenuated in the EAE+EX animals compared with EAE+ no-EX (*P<*0.001). 

***Aerobic training increases Ang-1 expression but attenuates the effects of EAE induction on Ang-1***

**Figure 4:**
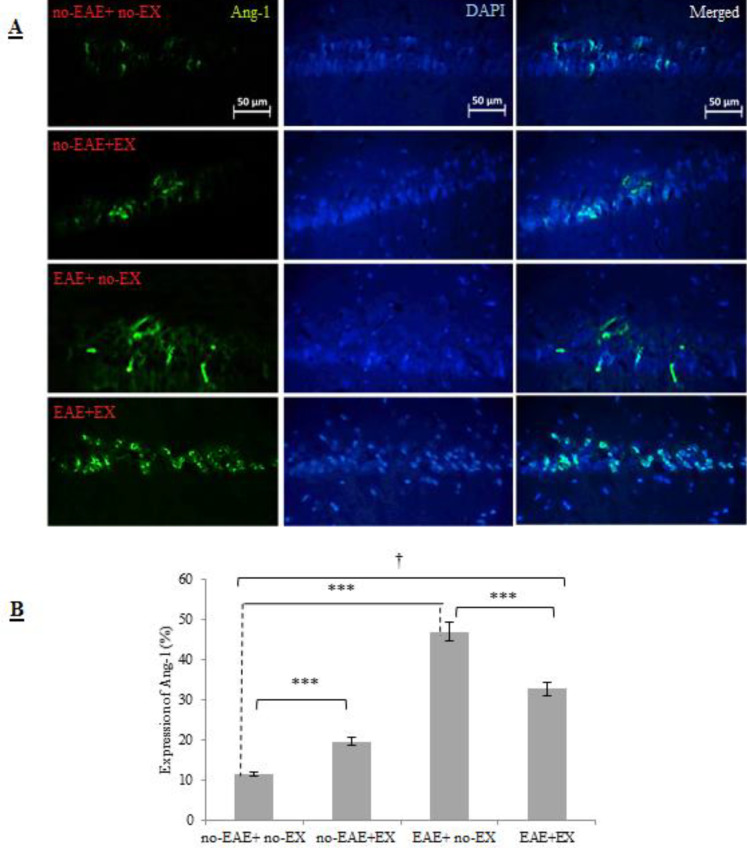
Expression level of hippocampal Ang-1 in four groups following six-week treadmill aerobic training. A) Microscopic staining of hippocampal Ang-1 protein and B) Graph of % expression of Ang-1 protein based on statistical analysis of microscopic images by using IMAGE J software. Section (B) also represents aerobic training increased Ang-1 expression and attenuated its expression induced by EAE induction. Ang-1, angiopoietin 1; no-EAE+ no-EX, the group without exercise training and without EAE induction; no-EAE+EX, exercise training group; EAE+ no-EX, experimental autoimmune encephalomyelitis group without exercise, EAE+EX, the combined experimental autoimmune encephalomyelitis + exercise group. Data are expressed as percentages. *** Indicates statistical significance at *P*<0.001; † Indicates significant difference among groups at *P*<0.001

EAE: experimental autoimmune encephalomyelitis; EX: exercise

Based on two-way ANOVA, a meaningful interaction was identified in Ang-1 expression as an agent in contributing to changed BBB integrity (*P<*0.001, effect size = 0.97, large). It implies that the effects of aerobic training and EAE induction were different on this marker. The results from the main effects signify that groups submitted to six-week aerobic training and those without training were significantly different in the rate of Ang-1 expression. There were statistically significant differences among the four groups in Ang-1 expression levels. The hippocampal levels of Ang-1 were significantly increased in animals of the no-EAE+EX group compared with the no-EAE+ no-EX group (*P<*0.001). As demonstrated in Figure 4, Ang-1 level was increased in EAE+ no-EX animals compared with the no-EAE+ no-EX animals (*P<*0.001). Importantly, Ang-1 decreased in EAE+EX group compared with EAE+ no-EX group ((*P<*0.001).

***Aerobic training down-regulates TJ proteins but restores their levels subsequent to EAE induction***

**Figure 5:**
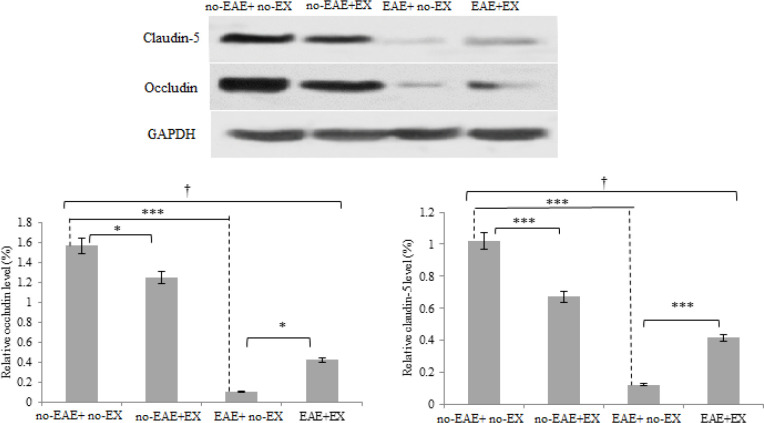
Tight-junction protein analysis by using western blot quantification. Relative expression of two tight-junction proteins of claudin-5 and occludin in four groups following six-week treadmill aerobic training. As the figure depicted aerobic training reduced both tight-junction proteins and also attenuated the reduction of both proteins induced by EAE induction. no-EAE+ no-EX, the group without exercise training and without EAE induction; no-EAE+EX, exercise training group; EAE+ no-EX, experimental autoimmune encephalomyelitis group without exercise, EAE+EX, the combined experimental autoimmune encephalomyelitis + exercise group. Data are expressed as percentages. * Indicates statistical significance at *P*<0.05; *** indicates statistical significance at *P*<0.001; † Indicates significant difference among groups at *P*<0.001

EAE: experimental autoimmune encephalomyelitis; EX: exercise

By comparing the relative levels of TJ proteins as main factors of BBB integrity by western blot technique, there was a significant interaction (*P<*0.001, effect sizes of 0.97 and 0.51 for claudin5 and occludin, respectively). The significant interaction in both markers demonstrates that aerobic training-mediated alterations of TJ proteins are different in groups of EAE induction. Furthermore, meaningful differences were obtained among the four groups. *Post hoc* test indicated that there were significant differences between no-EAE+ no-EX and EAE+ no-EX groups as well as between no-EAE+EX and no-EAE+ no-EX (*P<*0.001). As illustrated in Figure 5, significantly decremented changes in claudin-5 and occludin levels were found in EAE+ no-EX animals compared with the no-EAE+ no-EX group (*P<*0.001). Notably, there was a significant increase in claudin-5 and occludin levels for EAE+EX compared with EAE+ no-EX (*P<*0.001). Importantly, claudin-5 (*P<*0.001) and occludin (*P<*0.05) were significantly reduced in no-EAE+EX compared with no-EAE+ no-EX. 

**Figure 6:**
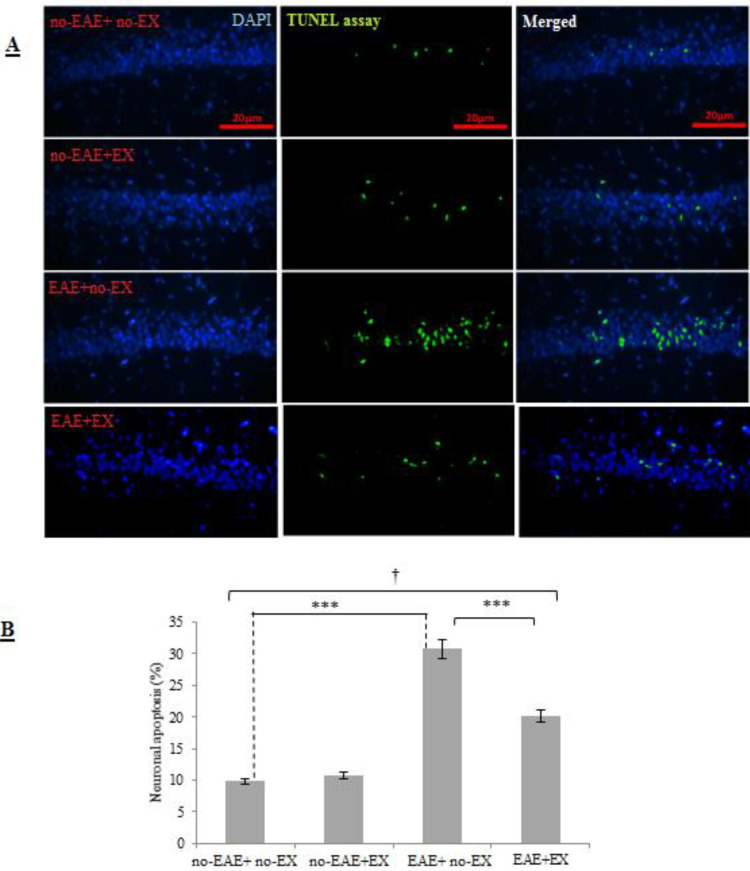
Percentage of hippocampal neuronal apoptosis (TUNEL+ cells) in four groups following six-week treadmill aerobic training. A) Microscopic staining of TUNEL+ cells and B) Graph of % neuronal apoptosis based on statistical analysis of microscopic images by using IMAGE J software. Section (B) also shows EAE induction increased neuronal apoptosis, and aerobic training attenuated EAE-induced neurodegeneration. no-EAE+ no-EX, group without exercise training and without EAE induction; no-EAE+EX, exercise training group; EAE+ no-EX, experimental autoimmune encephalomyelitis group without exercise, EAE+EX, combined experimental autoimmune encephalomyelitis + exercise group. Data are expressed as percentages. *** Indicates statistical significance at *P*<0.001; † Indicates significant difference among groups at *P*<0.001

EAE: experimental autoimmune encephalomyelitis; EX: exercise

According to the neuronal apoptosis results obtained from two-way ANOVA, there was a statistically significant EX × EAE interaction (*P<*0.001, large effect size= 0.94). It indicates the heterogeneous effects of exercise training and EAE induction on neuronal apoptosis. As illustrated in Figure 6, the groups had significant differences in neuronal apoptosis based on the TUNEL assay. According to the *post hoc* test, neuronal apoptosis was not significantly changed in no-EAE+EX compared with no-EAE+ no-EX (*P*= 0.271). There was an increased level of apoptosis in EAE+ no-EX animals compared with the no-EAE+ no-EX group (*P<*0.001), and the rate of apoptosis was significantly decreased in EAE+EX compared with the EAE+ no-EX group (*P<*0.001).

## Discussion

Our results demonstrated that the expression levels of GFAP and Ang-1 were increased following aerobic training, but neuronal apoptosis was not changed by the six-week training protocol. Moreover, the expression of TJ proteins down-regulated after being implemented in six-week aerobic training. On the other hand, progressive treadmill aerobic training reversed the effects of EAE induction on interest brain markers through diminishing astrocyte activation, Ang-1 levels, and neuronal apoptosis. Besides, aerobic training blocked the effects of EAE induction on TJ proteins, or rather, the aerobic training restored the levels of TJ proteins in animals with EAE induction. 

Parallel to our result, it has previously been reported that 3 and 6 weeks of treadmill running (30 min daily) increased astrocytosis in both brain areas of cortex and striatum (9). Exercise training, especially aerobic training, may protect BBB from dysfunction resulting from neuropathological diseases like MS, through increased astrocytosis. Induced astrocytosis strengthens the neurovascular unit (NVU) integrity which consists of neurons, vascular endothelium, and astrocytes (9). Increased energy demands and central and peripheral neurotrophic factors like BDNF, endothelial growth factor (EGF), FGF, IL-6, and adenosine during aerobic training might be primary reasons for this rise in GFAP expression (29). For example, increased energy and oxygen demands during aerobic exercise initiate some signals delivered by cerebrovascular structure to astrocytes for meeting neuronal cells. Upon receiving these signals, astroglia were afflicted with morphological and functional changes for meeting the neuronal demands through releasing the factors such as Ang-1 and vascular endothelial growth factor (VEGF), which are essential for angiogenesis. Persistent contribution in aerobic exercise is associated with new cerebrovascular formation and as a consequence astrocyte proliferation (astrocytosis) in involved brain areas to support the new vessel formation **(**9**)**. GFAP level is positively related to the progression of neurodegenerative diseases. In this regard, our findings signified that EAE induction causes reactive astrogliosis based on GFAP expression on astrocytes. Reactive astrocytes are associated with structural and functional changes which are followed by compromised BBB integration through loss of end-feet physical connection to endothelial structure (1). On this basis, reduction of GFAP can be regarded as a marker monitoring treatment efficiency. In this context, there is not enough evidence in light of GFAP expression changes after exercise training in MS. In parallel with our findings, the only animal study of MS by feeding cuprizone (CPZ) indicated that six-week exercise training reduces GFAP expression in the corpus callosum demonstrating astrogliosis attenuation (30). In this manner, aerobic training may enhance BBB integrity and consequently prevent exceeding immune cell infiltrations into the CNS (31). Ameliorating and reducing effects of exercise training on GFAP expression may be due to decreased inflammatory mediators by microglia, increased regulatory T cells (Tregs) in the CNS, and/or immune deviation from T-helper1 toward Tregs **(**1, 31**)**.

Additionally, Ang-1 is a neurotrophic factor secreted by neurons and most majority by glial cells, especially astrocytes with the major function on BBB-establishing endothelial cells (32). In accordance with our results, the previous studies on animal and human subjects have corroborated our findings (32, 34). This effect may contribute more integrity to NVU and BBB via up-regulation of the tight junction proteins (12) which thereby supply enough oxygen and energy to neuronal cells during neuronal activity (35, 36). There is an opacity related to the effect of exercise training on Ang-1 expression in various brain regions. The regulation of Ang-1 expression is affected by the inflammatory status of the microenvironment (37). In this context, our results suggested that inflammatory EAE condition triggered an up-regulation in Ang-1 expression. Increased astrocyte-derived Ang-1 expression suffers BBB more percolated in inflammatory microenvironment through down-regulating of TJ proteins and also attraction of immune cells to endothelial cells (13). The findings from our study identified that aerobic training mitigated the increased expression of hippocampal Ang-1, but it cannot return the level of Ang-1 to the baseline. To the best of our knowledge, this is the first report regarding the effects of exercise training on Ang-1 expression in CNS during EAE induction. Thus, it is suggested that reduced Ang-1 protein following aerobic training can attenuate EAE induced BBB permeability and consequent complications. 

The evidence in parallel with our findings regarding the exercise-induced changes of TJ proteins (claudin-5 and occludin) is scant. The previous evidence only investigated BBB permeability following exercise that was measurable by serum indicators such as S100β. In this way, Sharma *et al*. reported that forced swimming increases BBB permeability in certain brain areas and they ascribed this increase to serotonin and its receptors (38). It has been suggested that compromised BBB function induced by exercise is caused without existing structural changes (39). Consistent with our findings, evidence has been disclosed that exercise may increase BBB permeability which is attributed to hyperthermia, increased circulatory concentrations of ammonia, adrenaline, noradrenaline (5), increased inflammatory mediators (19), changes in some central neurotransmitters like serotonin and glutamate, excessive production of ROS, and increased production of lactate-mediated growth factors (5, 40). In this context, the majority of these compromising BBB factors are triggered by the increased metabolic and hypoxic stresses and also hemodynamic changes during progressive aerobic training. Increased metabolic stress in the brain during aerobic exercise causes ROS formation through electron leakage from the electron transfer chain (ETC) of mitochondria (41, 42). Brain components including cerebrovascular structure are extremely vulnerable to ROS and consequent oxidative stress. This sensitivity is ascribed to high containment of lipids and phospholipids, low anti-oxidant capacity, high metabolic rate, and a large amount of metals (41). Other mentioned factors activate the hypoxia-inducible factor-1 alpha (HIF-1α) which consequently binds to the promoters of vascular endothelial growth factor (VEGF)-A and endothelial nitric oxide synthase (eNOS) genes (43, 44). ENOS and VEGF-A are primarily expressed/released by endothelium and astrocytes, respectively (45, 46). Then, eNOS produces nitric oxide (NO) which in high extent concentration binds to other presented free radicals including superoxide for forming peroxynitrite. Peroxynitrite can induce membrane degeneration of endothelial cells via lipid peroxidation (47, 48). Importantly, it has been also suggested that astrocyte-produced VEGF-A can down-regulate TJPs by interaction on its receptors on BBB (49). On other hand, it has been reportedly identified that EAE induction engendered BBB permeability through down-regulation of these TJ proteins (10, 46). The probable mechanisms responsible for this down-regulation have been attributed to increased matrix metalloproteinases (MMPs) and glutamate excitotoxicity resulting from EAE induction (10, 46). Aerobic training, conversely, reduced/reversed the down-regulation of both TJ proteins induced by EAE induction. Coincidentally, some clinical evidence has shown a positive exercise training effect on BBB permeability via increasing oxidative capacity, reducing inflammatory factors which consequently prevent disease-induced tight-junction down-regulation (19). 

Exercise training is an intervention to prevent neuronal apoptosis and also has positive effects on neuronal plasticity and proliferating new neurons (15). In this context, our report is in parallel with antecedent reports, which showed no changes in neuronal apoptosis following exercise training (23). However, it has been also illustrated that neuronal apoptosis is increased in some diseases like MS (50), which is actually congruent with our results. Hyper-inflammation and excessive oxidative stress induced by EAE induction are probable mechanisms to trigger apoptotic processes (51). In any case, our findings in reversing the effects of exercise training on neuronal apoptosis in the inflammatory condition is comparable with the previous report performed by Jen *et al*.; they found that 30 min for 2-week swimming in EAE-induced rats caused a reduction in hippocampal neuronal apoptosis (50). Thus, aerobic training plays a preventive and modifying role in attenuating the apoptotic cells and cognitive impairment induced by MS. The mechanisms associated with endurance training in reducing EAE-induced neuronal apoptosis may include increased DNA damage capacity through promoting base excision enzymes and up-regulating BDNF expression as well as attenuating oxidative damage (52).

Admittedly, in the EAE group, the clinical scores initiated on day 7 after EAE induction and reached a higher level on day 14. Similar to our findings, a study conducted by Souza and coworkers indicated that one-week aerobic training delays the initiation clinical scores (19). This research group also showed that EAE induction caused a visible reduction in animal body weight, the result of whom in this regard was consistent with our finding. It may be concluded that early contribution in physical exercise, especially aerobic training, is more cost-effective to suggest to those who are at higher risk and also to individuals with MS disease. Importantly, it should be mentioned that caution is needed to ascribe all effects to exercise training since the severity of the EAE course can be influenced by fluctuations in sex hormones, especially in female strains during the menstrual cycle (53), and it is critically important to address this matter in prospective investigations. Besides, it appeared that the weight can be influenced by MS in individuals who are activated prior to suffering from a pathological condition like MS. Weight loss triggered by EAE induction is attributed to paralysis, reduced food intake, and high production of pro-inflammatory cytokines which affect the body systemically. 

The strength of the current report is that it evaluated the numerous parts involved in BBB integration in MS disease, including astrocytes, Ang-1 as a factor released by astrocytes, TJ proteins, and neuronal apoptosis as a consequence of increased BBB permeability. Among the shortcomings of our study that might be helpful to improve the value and accuracy of current research are not evaluating matrix metalloproteinases (MMPs), vascular endothelial growth factor-A (VEGF-A), IL-1β, and microglial reactivation. Another shortcoming is that we did not examine the changes in astrocytes value and their end-feet. Besides, other brain areas such as the spinal cord and brain stem among others may be influenced by the current procedure of EAE induction; our focus on hippocampal tissue is probably considered as a limitation in the present study. 

## Conclusion

Our results suggest that aerobic training in normal conditions increased the expression of hippocampal GFAP (astrocytosis) and Ang-1 proteins, while down-regulating the expression of TJ proteins. Under inflammatory disease conditions of EAE induction, aerobic training preserved BBB integrity through reducing astrogliosis and Ang-1 as well as reversing EAE-induced TJ protein down-regulation. Besides, six-week aerobic training provided a protective role to neurons via attenuation of neuronal apoptosis. 

## Authors’ Contributions

All authors contributed to the study design and revised the final draft. OR Collected and analyzed the data and wrote the first draft. AP Supervised the project and revised and commented on the draft. IR Administered the project and supervised the creation of the animal model. NP Analyzed the data and supervised the project conduction, gave scientific consultation, and revised and commented on the first draft. SER Supervised the project conduction. RWM Provided critical insight on data analysis and the drafts of the paper. 

## Funding

This research did not receive any specific grant from funding agencies in the public, commercial, or not-for-profit sectors.

## Conflicts of Interest

The authors declare that there are no conflicts of interest.
